# An Avatar-Led Digital Smoking Cessation Program for Sexual and Gender Minority Young Adults: Intervention Development and Results of a Single-Arm Pilot Trial

**DOI:** 10.2196/30241

**Published:** 2021-07-30

**Authors:** Jaimee L Heffner, Noreen L Watson, Edit Serfozo, Megan M Kelly, Erin D Reilly, Daniella Kim, Kelsey Baker, N F N Scout, Maria Karekla

**Affiliations:** 1 Fred Hutchinson Cancer Research Center Seattle, WA United States; 2 Veterans Affairs Bedford Healthcare System Bedford, MA United States; 3 University of Massachusetts Medical School Worcester, MA United States; 4 University of Washington Seattle, WA United States; 5 National LGBT Cancer Network Providence, RI United States; 6 University of Cyprus Nicosia Cyprus

**Keywords:** LGBT, embodied agent, tobacco cessation, nicotine dependence, user-centered design, avatar, digital health, minority, young adult, teenager, smoking, cessation, intervention, development, pilot trial

## Abstract

**Background:**

Sexual and gender minority young adults have a high prevalence of smoking and unique barriers to accessing tobacco treatment.

**Objective:**

To address these challenges as well as their preferences for sexual and gender minority–targeted interventions and digital programs, we developed and evaluated the acceptability, preliminary efficacy, and impact on theory-based change processes of an acceptance and commitment therapy–based digital program called Empowered, Queer, Quitting, and Living (EQQUAL).

**Methods:**

Participants (n=22) of a single-arm trial conducted to evaluate the program were young adults, age 18 to 30 years, who self-identified as sexual and gender minority individuals and smoked at least one cigarette per day. All participants received access to the EQQUAL program. Participants completed web-based surveys at baseline and at a follow-up 2 months after enrollment. We verified self-reported smoking abstinence with biochemical testing; missing data were counted as smoking or using tobacco.

**Results:**

For young adults who logged in at least once (n=18), the mean number of log-ins was 5.5 (SD 3.6), mean number of sessions completed was 3.1 (SD 2.6), and 39% (7/18) completed all 6 sessions. Overall, 93% of participants (14/15) were satisfied with the EQQUAL program, 100% (15/15) found it easy to use, and 100% (15/15) said it helped them be clearer about how to quit. Abstinence from smoking or using tobacco was confirmed with biochemical testing for 23% of participants (5/22). Both quantitative and qualitative results suggested a positive overall response to the avatar guide, with areas for future improvement largely centered on the avatar’s appearance and movements.

**Conclusions:**

Treatment acceptability of EQQUAL was very promising. The rate of abstinence, which was biochemically confirmed, was 3 times higher than that of the only other digital program to date that has targeted sexual and gender minority young adults and 6 to 13 times higher than those of nontargeted digital smoking interventions among sexual and gender minority young adults. Planned improvements for the next iteration of the program include making the avatar’s movements more natural; offering multiple avatar guides with different on characteristics such as race, ethnicity, and gender identity from which to choose; and providing a support forum for users to connect anonymously with peers.

## Introduction

The prevalence of tobacco use among sexual and gender minority young adults is twice as high as that among non–sexual and gender minority young adults across all tobacco products: 29% vs 15% for cigarettes and 8% vs 4% for both electronic nicotine delivery systems and noncigarette tobacco, respectively [1]. Compounding their risk for tobacco-related diseases, sexual and gender minority young adults also encounter unique barriers to accessing treatment. Almost half of sexual and gender minority young adults lack health insurance to pay for traditional treatments such as counseling and pharmacotherapy [2], and there is substantial underuse of existing public health interventions such as tobacco quitlines among this group [3]. There is tremendous unrealized opportunity to reach these young tobacco users via digital means, because they are high adopters of technology [4] and would benefit from the availability of a high-reach, no-cost intervention due to the multiple barriers they face in accessing traditional forms of treatment. However, only one study [5], published recently, tested a targeted digital intervention—a professionally moderated intervention delivered via Facebook—that was specifically for sexual and gender minority young adults. Although the study showed low biochemically confirmed quit rates overall, it demonstrated the potential efficacy of sexual and gender minority–targeted content, with quit rates for the targeted social media intervention (7%) almost double that of the nontargeted social media intervention (4%) [5].

To address the need for an engaging, effective, and accessible treatment approach for sexual and gender minority young adult tobacco users, we engaged in a user-centered design process to develop a digital cessation program targeted for this group and subsequently conducted a single-arm pilot trial to evaluate its acceptability (primary outcome) and preliminary efficacy (secondary outcome) for motivating and supporting smoking cessation. Acceptability, as well as intervention impact on change processes and outcomes (ie, efficacy), are standard benchmarks in intervention development [6]. Together, they help provide proof-of-concept evidence that users will engage with the intervention and that it can impact theory-based change mechanisms and, ultimately, the outcome of interest. The purpose of this study was to evaluate components of the novel program requiring further refinement and to plan a future randomized clinical trial as a next step in treatment development and evaluation.

## Methods

### Part 1: User-Centered Design of EQQUAL

The Empowered, Queer, Quitting, and Living (EQQUAL) program is a cultural, linguistic, and sexual and gender minority–targeted adaptation of Flexiquit, a web-based acceptance and commitment therapy (ACT) program designed for young adults at all stages of readiness to quit tobacco use [7]. In addition to focusing on increasing acceptance of smoking triggers and facilitating values-guided action as cessation treatment mechanisms, ACT also teaches generalizable emotion regulation skills that can increase resilience and buffer minority stress [8] among sexual and gender minority young adults. This generalizability stems from a transdiagnostic focus on building psychological flexibility—defined as willingness to experience the full range of emotional, physical, and cognitive experiences without trying to change them and to do things that are difficult in service of one’s values [9,10]. EQQUAL also employs another exciting innovation in digital tobacco treatment: an avatar guide designed to make the program more engaging and effective. The original avatar-led Flexiquit program (not sexual and gender minority–targeted) showed great promise in motivating cessation in a pilot randomized controlled trial with young adults in Cyprus, with a 29% posttreatment quit rate for Flexiquit versus 11% for the waitlist [7]. Importantly, the majority (65%) of participants were in the precontemplation or contemplation stages of change, demonstrating the program’s utility for smokers who are at lower levels of quit readiness [7].

To adapt the original version of the program, which was created for the general population of young adults in Cyprus, to one for a US young adult population self-identifying as sexual and gender minority, 3 major modifications were needed: (1) translation from Greek to English, (2) adaptation of content for US culture (eg, providing examples and metaphors that are more relevant to life in the US), and (3) adaptation of content to sexual and gender minority young adults. After translation, which occurred from August to September 2018, we followed a user-centered design process to adapt the program for the US culture and to a sexual and gender minority young adult population. This included 2 rounds of preliminary user testing (n=7 participants; 2 self-identified as female, 4 self-identified as male, and 1 did not self-identify as male, female, or transgender; 3 self-identified as bisexual and 4 self-identified as gay or lesbian; 2 self-identified as non-Hispanic White, 1 self-identified as non-Hispanic Asian, 1 as self-identified as non-Hispanic Asian and White, and 3 did not provide their race and ethnicity) and a diary study in which participants used the program for 1 week and provided feedback about it via daily diary entries and a final interview (n=8; 2 self-identified as female, 1 self-identified as male; 5 self-identified as transgender or nonbinary; 1 self-identified as bisexual, 2 self-identified as gay, 2 self-identified as queer, and 2—who self-identified as gender minority—did not provide their sexual orientation; 3 self-identified as non-Hispanic White, 2 self-identified as non-Hispanic Asian, and 3 did not provide their race and ethnicity).

**Figure 1:**
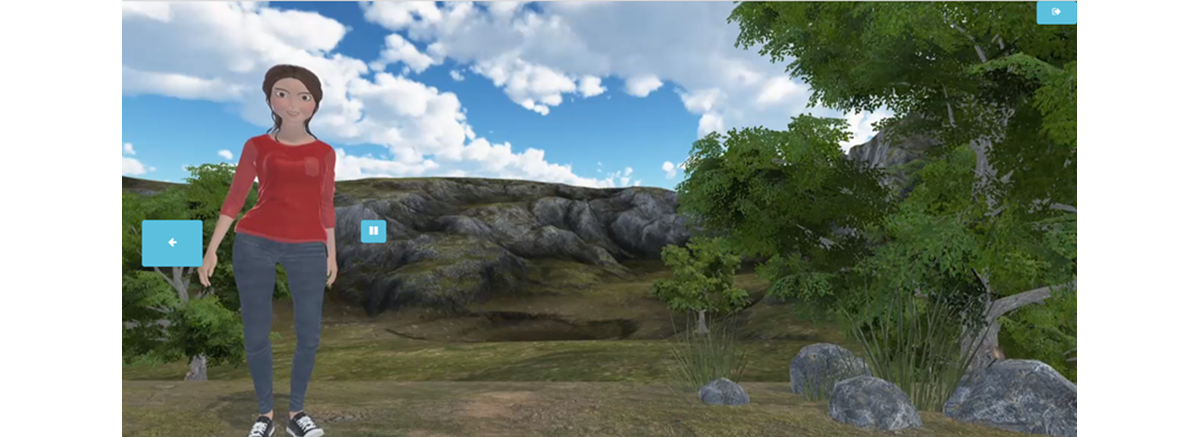
Avatar from original, nontargeted Flexiquit.

A major focus of the user-centered design work to create EQQUAL was updating the appearance, voice, and persona of the avatar guide. The most recent version of the avatar used in the original, nontargeted Flexiquit program was presented as a young woman (Figure 1). As part of the testing, we evaluated users’ reactions to this avatar as well as to several other potential program guides, including both human and nonhuman representations. Users unanimously preferred a human guide, with desired qualities being inspiring, fun, and sensitive. Users also indicated that they preferred having a visual representation of the program guide, rather than just a voice, as something concrete that they could count on consistently while they completed the program. Visually, users preferred cartoon-like over realistic-looking avatars. They also preferred avatars that appeared to be androgynous or gender ambiguous. Among the possible human representations presented to users, the previous Flexiquit avatar was not preferred. Users agreed that the avatar should have a backstory and share it and that the tone of the avatar should be conversational and informal. Participants wanted to be able to hear and read the content simultaneously. Several options for voices of the avatar were presented, and a preferred voice was selected on the basis of user testing, which was the voice of a young adult woman. Participants also expressed a desire for an avatar that could be customized or selected based on their ability to identify with it or be inspired by it. With the exception of the customizable avatar, which was cost-prohibitive for this phase of development, all of these user preferences were incorporated into the design of the EQQUAL program.

**Figure 2:**
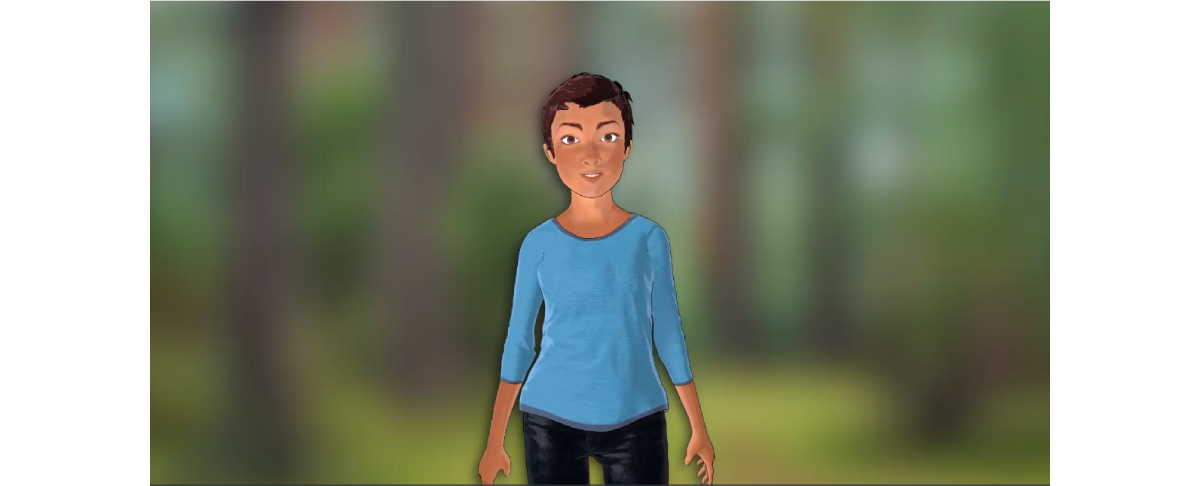
New sexual and gender minority–targeted Empowered, Queer, Quitting, and Living (EQQUAL) program avatar.

Aside from the new appearance and backstory of the EQQUAL avatar (Figure 2), user-centered design for sexual and gender minority targeting included development of user stories about sexual and gender minority young adults sharing their motivations and successes in quitting smoking. These stories were written by the research team and evaluated during user testing. During the interviews, users also noted a preference for a social component to the program that would allow users to connect anonymously, such as a quit buddy or support group, as well the ability to give and receive kudos for reaching smoking-related milestones. Similar to avatar customization, this functionality was outside of the scope for this phase but is a planned direction for future versions.

In accordance with stated user preferences of accessing the program on a mobile device, we developed EQQUAL as a responsive mobile-optimized website designed to be used primarily on mobile and tablet devices. Programming was completed in May 2020, at which point we launched a single-arm pilot trial to evaluate its acceptability and preliminary efficacy for cessation and impact on theory-based change mechanisms.

### Part 2: Single-Arm Pilot Trial

#### Participants

Eligibility criteria were individuals (1) between 18 and 30 years of age who (2) self-identified as sexual or gender minority (ie, a sexual orientation other than straight, gender that doesn’t match sex assigned at birth, or both); (3) resided in the United States; (4) smoked at least 1 cigarette per day over the past 30 days; (5) had at least weekly internet access and were willing and able to stream video online during the 2-month study period; (6) used text messaging; (7) were not engaged in any other smoking cessation treatment at the time of screening, including pharmacotherapies or behavioral counseling, but excluding e-cigarettes or other vaping devices, which are not approved by the US Food and Drug Administration for smoking cessation; and (8) were comfortable reading, writing, and speaking in English.

#### Procedures

All study procedures were reviewed and approved by the Scientific Review Committee and the Institutional Review Board of the Fred Hutchinson Cancer Research Center.

Participants were recruited online between May and June 2020 via Facebook and Craigslist advertisements and directed to a screening survey administered via REDCap (Vanderbilt University). To prevent potential fraudulent participation, study staff reviewed the responses of individuals who were potentially eligible for duplicate entries and email addresses were verified. Individuals who were eligible were sent an email indicating their eligibility that required a reply. Individuals who did not reply after 3 attempts (2 reminders were sent over a 14-day period) were no longer contacted for potential recruitment and were sent an email with alternative quit-smoking resources (eg, referrals to 1-800-QUIT-NOW to reach their state quitline and the National Cancer Institute’s Smokefree web and mobile app programs).

Individuals who responded to the email were sent another email (and up 2 reminders over a 14-day period, as needed) inviting them to complete a confidential, secure online survey to provide informed consent and complete the baseline assessment. After submitting the baseline assessment, participants received an email indicating that they were enrolled in the study that provided log-in credentials for EQQUAL and information about technical support, study payment, and the follow-up survey. Individuals who did not consent or complete the online enrollment process within 14 days were sent an email notifying them that they were not enrolled; the email provided them with the smoking cessation resources mentioned above.

Two months after enrollment, participants were sent an email inviting them to complete a web-based follow-up survey. A text message was sent on the same day as the email survey invitation to alert participants that the survey would be arriving via email that day. To maximize data retention, participants were sent up to 2 email reminders within 9 days indicating that the follow-up survey was available. Participants who did not complete the survey were called once daily for 5 days and were given the opportunity to complete the follow-up survey by phone. A final attempt was made—a postcard with just 2 follow-up questions (ie, the primary acceptability and efficacy outcome questions) was sent out to those who had not responded by the fifth phone call. Baseline and follow-up survey data were collected and stored in a secure REDCap database.

Participants were compensated US $25 for completing the follow-up survey. To maximize data retention, respondents received a $10 bonus if they completed the survey online within 24 hours of the initial email. Participants who self-reported smoking abstinence were asked to submit saliva cotinine test results and received an additional $25 for doing so. Thus, participants could receive up to $60 in total.

#### Assessments

The baseline survey collected information on demographic characteristics, use of alcohol and electronic cigarettes, and smoking history and current smoking behaviors, including the Fagerström Test for Nicotine Dependence score [11]. To assess sexual and gender minority identity, we used 3 items based on consensus recommendations [12-14]. Two gender minority items focused on sex and gender identity: “What was your assigned sex at birth, on your original birth certificate?” (response options: male, female); “What is your current gender identity (check all that apply)?” (response options: man/male, woman/female, trans male/trans man, trans female/trans woman, genderqueer/gender nonconforming, different identity, not sure). To assess sexual minority identity, participants were asked: “Do you think of yourself as: (check all that apply):” (response options: straight (heterosexual), bisexual, gay, lesbian, queer, different than listed, not sure). We considered participants whose current gender identities differed from their birth sex or who answered that they were trans male/man, trans female/woman, genderqueer/gender nonconforming, different, or not sure as gender minority. We considered participants who gave any response to the sexual orientation question other than straight/heterosexual as sexual minority. Since recruitment occurred during the early period of the COVID-19 pandemic, we also included 1 question on the baseline survey (ie, “Since learning about the coronavirus—also known as COVID-19—has your smoking decreased, increased, or stayed the same?”) and 1 question on the follow-up survey (ie, “Since you started this study, to what extent has the coronavirus—also known as COVID-19—played a role in motivating you to reduce or quit smoking?” Response options were “not at all,” “slightly,” “somewhat,” “moderately, and “extremely”) to assess the impact of COVID-19 on baseline smoking and motivation to quit during the treatment period.

Primary treatment acceptability endpoints were (1) server-recorded number of log-ins and number of sessions completed during the 2-month study period, and (2) treatment satisfaction, which was assessed using 12 study-specific items on the follow-up survey (eg, “Overall, how satisfied were you with EQQUAL?”). Secondary endpoints were (1) biochemically confirmed 7-day point prevalence smoking abstinence at 2 months, and (2) changes in readiness to quit smoking from baseline to follow-up 2 months later assessed via the 11-point Contemplation Ladder [15], which ranges from 0 (“No thought about quitting”) to 10 (“Taking action to quit (eg, cutting down, enrolling in a program)”. Self-reported 7-day point prevalence abstinence (PPA) from smoking was assessed via the question: “When was the last time you smoked, or even tried, a cigarette?” Responses of “8-30 days ago” and “over 30 days ago” were classified as 7-day PPA. All participants who self-reported 7-day PPA at follow-up and no other nicotine use in the past 7 days were sent Alere iScreen saliva kits via overnight mail to verify their smoking status. The iScreen offers qualitative detection of cotinine in saliva at a cut-off level of 30 ng/mL. Participants were sent instructions to complete the test at home and submit test results via a photo of the completed test uploaded in a REDCap form.

Changes in psychological flexibility, as the theory-based mechanisms of change, from baseline to 2 months were exploratory endpoints. This included (1) changes in acceptance of smoking triggers (emotional and physical subscales of the adapted Avoidance and Inflexibility Scale [16,17], 18 items), (2) overall psychological flexibility (24-item short version of the Multidimensional Psychological Flexibility Inventory [18], which has 2 composite scores representing psychological flexibility and psychological inflexibility), and (3) valued living (10-item Valuing Questionnaire [19], which has 2 subscales representing values progress, 5 items, and values obstruction, 5 items).

We also explored acceptability of the EQQUAL avatar, called Jen, at follow-up 2 months after enrollment using the Agent Persona Inventory [20], which contains 25 items rated on a 1 (strongly disagree) to 5 (strongly agree) scale, with 4 subscales: facilitating learning (10 items; eg, “Jen kept my attention”), credible (5 items; eg, “Jen was knowledgeable”), human-like (5 items; eg, “Jen showed emotion”), and engaging (5 items; eg, “Jen was expressive”). We also included a set of items assessing the avatar’s attributes, drawing in part from items on the Robotic Social Attributes Scale [21]. On these items, the avatar’s attributes were rated on a 1 (definitely does not describe Jen) to 9 (definitely describes Jen) scale and included the following: pleasant, likable, agreeable, trustworthy, sincere, supportive, relatable, credible, scary, strange, awkward, and judgmental. We also included 4 open-ended, study-specific questions to assess acceptability of the avatar: (1) “Based on your experiences, what were the least important or least useful parts of Jen?” (2) “Based on your experiences, what were the most important or most useful parts of Jen?” (3) “What, if anything, would you change about Jen? Why?” (4) “What additional feedback would you like to provide us about the avatar in the program?”

### EQQUAL intervention

Consistent with the original, nontargeted program, EQQUAL contained 6 sessions designed to be completed in order, with a minimum of 3 days between sessions and automated pacing and prompting from the program. Sessions took approximately 10 to 30 minutes to complete. A single text message reminder was used to prompt availability of the next session. Session 1 PDF handouts were emailed to participants 2 days after enrollment, along with instructions for requesting technical support. At the end of the program, participants were sent an email with session handouts.

Content followed the ACT treatment model [9,22]. Session 1 introduced the avatar guide Jen who provided an overview of the program and shared their own story of quitting. Users completed an interactive game to identify personal values guiding quitting and reviewed stories from other sexual and gender minority young adults who quit smoking. Session 2 focused on trigger awareness through interactive questions, graphs, pictures, and experiential exercises and metaphors, and it introduced the ACT concept of creative hopelessness—recognizing that efforts to control thoughts, feelings, or sensations related to smoking can be counterproductive. Session 3 completed the topic of creative hopelessness and introduced cognitive defusion—psychological distancing from thoughts—as an alternative to thought control as a means of achieving goals and living a valued life. Session 4 completed the topic of cognitive defusion, encouraged setting a quit date in the subsequent week, and prompted users to practice defusing from thoughts (eg, “I won’t be able to quit”) as part of quit planning. Session 5 started with a reflection on the past week’s successes and difficulties, introduced acceptance and willingness to have unwanted thoughts and emotions as a means of handling smoking triggers, and covered relapse prevention via self-compassion and recommitment to quitting. Session 6 also started with a reflection on the past week’s successes and difficulties, reviewed content from previous sessions, and ended with a video emphasizing the importance of letting go of the need to control internal experiences such as feelings, sensations, and thoughts as a means to achieve goals. EQQUAL also included information about US Food and Drug Administration–approved smoking cessation medications and encouraged users to consider using a medication to support quitting. EQQUAL was accompanied by daily text messages that provided (1) motivational messages, (2) new session availability prompts, and (3) reminders of information discussed in the program.

**Figure 3:**
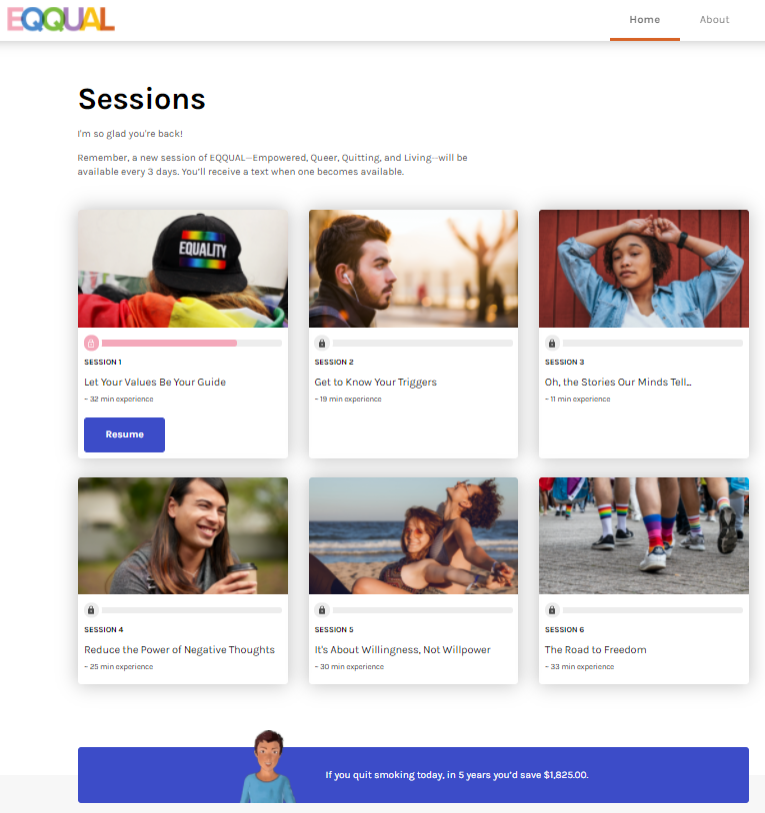
Home page screenshot.

**Figure 4:**
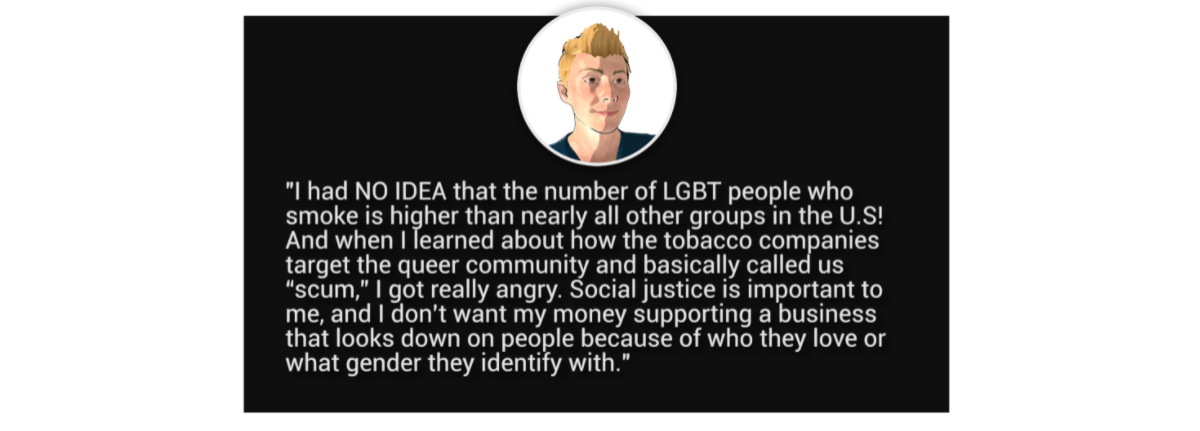
Sexual and gender minority–targeted quit story.

**Figure 5:**
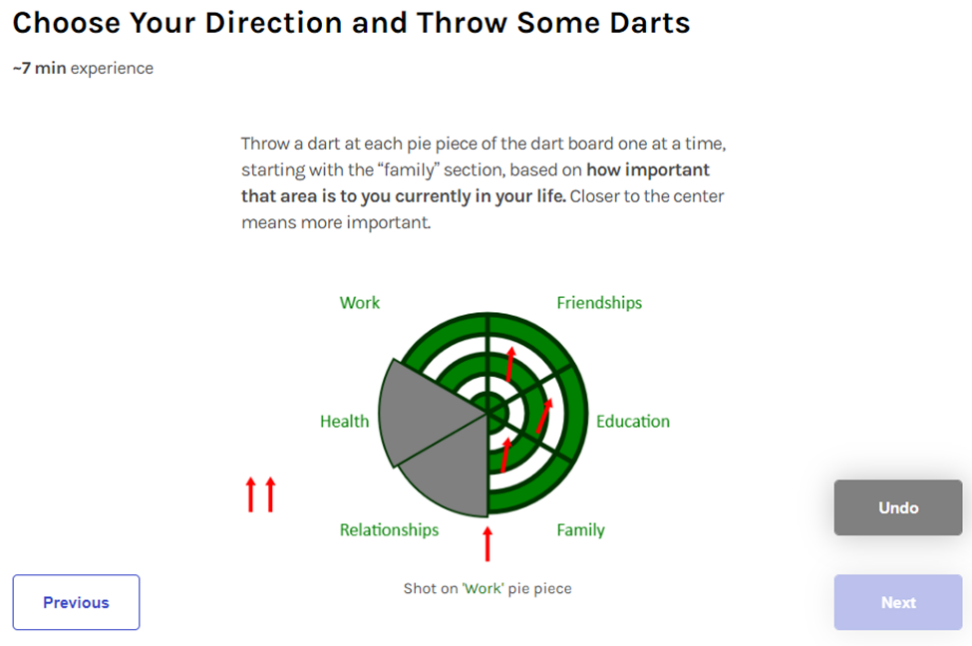
Interactive exercise screenshot.

The sexual and gender minority–targeted treatment content in EQQUAL focused on (1) unique motivations for quitting among sexual and gender minority individuals, such as the desire for freedom to live one’s life openly (eg, not having to hide smoking from family, as sexual and gender identity may have been hidden); (2) tools for coping with minority stress and its mental health sequelae (eg, evidence-based ACT skills for depression and anxiety); (3) self-compassion exercises focused on managing internalized shame, which can serve as a trigger for tobacco use; and (4) targeted videos and infographics describing the impact of tobacco use on the LGBTQ+ community. The appearance and backstory of the EQQUAL avatar guide was also designed to suggest that the avatar identified as a sexual and gender minority young adult (Figure 2). Figure 3 shows the program home screen (desktop view), along with examples of the sexual and gender minority–targeted quit stories (Figure 4) and interactive games to promote engagement with the program (Figure 5).

### Analysis

#### Sample Size

Because pilot trials differ from efficacy trials in their aims and scope, techniques that are employed for sample size determination in an efficacy trial (ie, power analysis) are not appropriate for a Stage 1 pilot trial [23]. The stage model of behavioral treatment development suggests that pilot treatment development trials should include approximately 15 to 30 participants per arm to test feasibility [23]. A sample size of 25 was chosen on the basis of this recommendation. Because this is a single-arm pilot trial, this study is not designed for power to detect statistically significant changes in response to treatment. However, we planned to preliminarily and descriptively assess the outcomes of the intervention to inform further refinements of EQQUAL and obtain the information necessary to optimize the study design in preparation for a subsequent Stage 2 efficacy trial.

#### Statistical Analysis Plan

Given the pilot nature of the study, all statistical analyses are descriptive. Frequencies (for categorical variables) or means and standard deviations (for continuous variables) are reported for all endpoints, including change scores. Categorical ratings for treatment satisfaction questions were dichotomized as useful (“somewhat,” “mostly,” or “very”) versus lower ratings based on a priori analysis plans (clinicaltrials.gov, NCT04194918). For smoking abstinence outcomes, we report results (1) including only participants who provided data on smoking abstinence at follow-up (complete-case) and (2) including all participants and imputing missing smoking data as nonabstinence.

## Results

### General

As planned, we enrolled 25 participants, but we excluded 3 participants postenrollment due to suspected fraudulent behavior (eg, providing different dates of birth at baseline and follow-up), leaving a final sample of 22 (Multimedia Appendix 1). Of these 22 participants, all identified as sexual minority (n=22, of whom 13 self-identified as bisexual, 3 self-identified as gay, and 6 self-identified as lesbian; 5 selected “queer” in addition to one of the other options) and a portion also self-identified as gender minority (n=7, of whom 3 self-identified as a trans man, 3 self-identified as genderqueer, and 1 self-identified as not sure). Over half (12/22; 55%) reported vaping as well as smoking. Ages ranged from 18 to 30, with a mean of 23.5 (SD 3.9). Over one-quarter (6/22; 27%) self-identified as racial minorities (n=1 self-identified as American Indian/Alaska Native, n=3 self-identified as Asian, n=2 self-identified as more than one race). The majority were employed (16/22; 73%) or in school (4/22; 18%) and were in a committed relationship (14/22; 64%). Average number of cigarettes smoked per day was 7.6 (SD 9.5), with a mean Fagerström Test for Nicotine Dependence score of 3.9 (SD 2.3). Baseline readiness to quit smoking ranged from 0 to 10 on the Contemplation Ladder, with a mean of 6.2 (SD 2.7). Regarding the impact of the COVID-19 pandemic on participants’ smoking, at baseline, the majority of participants (12/22; 54.5%) indicated that their smoking had stayed the same, 7/22 (31.8%) indicated that it increased, and 3/22 (13.6%) indicated that it had decreased. At follow-up, participants reported the extent to which COVID-19 played a role in motivating them to reduce or quit smoking as not at all (3/17; 17.6%), slightly (6/17; 35.3%), somewhat (2/17; 11.8%), moderately (3/17; 17.6%), or extremely (3/17; 17.6%).

### Design Feasibility

We screened 118 individuals over a 1-month period to enroll 22 participants. Recruitment occurred via Facebook (61% of those screened) and Craigslist advertisements (39%). Seventeen participants completed the follow-up survey (77% data retention). All 17 completed the survey online; 8 participants completed the survey within 24 hours of receiving the link, 3 participants completed the survey after 1 or 2 email reminders, and 6 participants completed the survey after 2 email reminders and 1 or more phone call reminders. Six participants who self-reported 7-day PPA from smoking and indicated using no other sources of nicotine during those 7 days were sent a cotinine kit, and all 6 (100% adherence) submitted a photo of the completed test as requested. One participant reported abstinence from cigarettes but use of other nicotine-containing products and was not sent a cotinine test kit.

### Primary Endpoint

Among those with at least 1 log-in (n=18), the mean number of log-ins was 5.5 (SD 3.6), mean sessions completed was 3.1 (SD 2.6), and 39% of participants (7/18) completed all 6 sessions. Among follow-up respondents who provided satisfaction ratings (n=15), 93% (14/15) rated EQQUAL as useful, 93% (14/15) reported being satisfied with the program, and 87% (13/15) said they would recommend it to a friend. Considering specific program components, 71% (10/14) felt that text messages were useful and 93% (14/15) reported that PDF handouts (eg, health benefits of quitting, smoking and the LGBTQ+ community) were useful. All but 1 participant (93%; 14/15) said they felt as though the program was made for them. All participants (15/15; 100%) said the program was easy to navigate, 100% (15/15) reported that they felt more clear about how they might quit as a result of using the program, and 93% (14/15) said it gave them new ways of looking at quitting.

### Secondary Endpoints

Biochemically confirmed 7-day PPA was 22.7% (5/22) with missing data imputed as continued smoking (missing=smoking), and 31.3% (5/16) using the complete-case method. Self-reported 7-day PPA was 31.8% (7/22) in the missing=smoking analysis and 41% (7/17) in complete-case analyses. Participants also showed an overall increase in readiness to quit, averaging a 2.1-point increase (SD 3.0) on the 11-point Contemplation Ladder from baseline to 2 months.

To understand the relationship between smoking abstinence and “dosage” received of the EQQUAL program, we descriptively examined, as a post hoc analysis, differences in program usage between those participants who achieved biochemically confirmed, missing=smoking 7-day PPA (n=5) to those who didn’t (n=17). Smoking abstainers had numerically higher program usage metrics than nonabstainers, including number of log-ins (mean 7.2, SD 3.9 for abstainers; mean 3.7, SD 3.6 for nonabstainers), number of sessions completed (mean 4.8, SD 2.7 for abstainers; mean 1.8, SD 2.3 or nonabstainers), and completion of all 6 sessions (80% of abstainers; 18% of nonabstainers).

### Exploratory Endpoints

Exploration of changes in values-based action on the Valuing Questionnaire [19]) showed a modest increase in Values Progress (mean +2.9, SD 4.4) and decrease in Values Obstruction (mean –3.3, SD 7.3). Acceptance of smoking triggers on the Avoidance and Inflexibility Scale [16] showed minimal average change from baseline to follow-up (mean +0.2, SD 0.8 for the emotions subscale score, mean –0.03, SD 0.9 on the physical sensations scale). On the Multidimensional Psychological Flexibility Inventory, the change in the psychological flexibility composite score from baseline to follow-up was + 0.3 (SD 0.8), and the change in the psychological inflexibility composite score was –0.3 (SD 1.2). In sum, the mean changes in the ACT-based measures of psychological flexibility were almost all in the hypothesized direction (ie, representing increased psychological flexibility and decreased inflexibility), albeit with low overall magnitude.

On the Agent Persona Inventory, all subscale scores suggested positive overall impressions of the avatar: facilitating learning (mean 41.9, SD 9.8, on a scale of 10-50), credible (mean 20.7, SD 5.2, on a scale of 5-25), human-like (mean 19.7, SD 5.1, on a scale of 5-25), and engaging (mean 20.4, SD 5.1, on a scale of 5-25). On items where participants rated the avatar’s attributes on a 1 to 9 scale from “definitely does not describe Jen” to “definitely describes Jen,” all of the positive attributes were rated highly: pleasant (mean 7.4, SD 2), likable (mean 7.5, SD 2), agreeable (mean 7.5, SD 2), trustworthy (mean 7.3, SD 2.1), sincere (mean 7.7, SD 2), supportive (mean 7.7, SD 2), relatable (mean 7, SD 2.3), and credible (mean 7, SD 2). Negative attributes all had lower scores: scary (mean 1.8, SD 1.4), strange (mean 2.4, SD 2), awkward (mean 2.5, SD 2.3), and judgmental (mean 2.3, SD 2.6).

Qualitative responses confirmed and extended the quantitative findings as well as suggesting specific areas for avatar improvement. Overall, we saw evidence that efforts to present Jen as human-like, supportive, nonjudgmental, and credible were effective. For example,

She encouraged me when I thought I couldn’t quit smoking.

...[I liked] how she seemed very intelligent.

She made it feel like a real person was there.

...[I liked] her approachable, nonjudgmental tone.

One participant even reported of Jen,

I’m in love with her.

When asked what they would change about Jen, 2 dominant themes emerged: (1) making the body movements more natural

She just kinda moved a little awkwardly

and (2) the need for the participant to be able to see themselves in the avatar’s appearance and voice

She should be a person of color so I can relate to her more.

## Discussion

The aim of this work was to develop and evaluate the acceptability and preliminary efficacy of an avatar-led digital smoking cessation program targeted for sexual and gender minority young adults. The product of our user-centered design work, the EQQUAL program, demonstrated strong acceptability and efficacy in a single-arm pilot trial with 22 sexual and gender minority young adults. For example, 93% of participants (14/15) were satisfied with the EQQUAL program, 100% (15/15) found it easy to use, and 100% (15/15) said it helped them be clearer about how to quit. Both quantitative and qualitative results suggested a positive overall response to the avatar, with areas for future improvement largely centered on the avatar’s appearance and movements. Regarding treatment utilization, including only those who logged in at least once (n=18), participants logged in an average of 6 times and completed an average of 3 sessions, with 39% completing all 6 sessions. This suggests that there is room for improvement in keeping users engaged with the program as a means of enhancing outcomes [24]. Indeed, our post hoc analysis suggested that there may be a relationship between engagement and outcomes given that 80% (4/5) of the participants who quit smoking completed all 6 sessions compared with only 18% (3/17) of the participants who did not quit. In addition to avatar improvements (eg, making the avatar’s body movements more natural) and customization options (eg, offering choice of avatars with varying gender expression, race, ethnicity, etc), in future iterations of the program, we also plan to implement a social feature that was suggested by users in the initial user-centered design work but was out of scope for the present work. Social connectedness has been demonstrated to increase engagement in other digital programs [25] and was associated with better engagement and abstinence outcomes in an online tobacco treatment program [26,27]. In addition, to prompt continued program use, we plan to include multiple session availability reminders rather than relying on a single reminder when a new session becomes available.

Compared to the only other targeted digital intervention for sexual and gender minority young adults—a professionally moderated Facebook intervention—the self-guided EQQUAL program’s biochemically confirmed quit rate was over 3 times higher (22.7% vs 7.1%) [5]. It is also 6 to 10 times higher than biochemically confirmed quit rates for the nontargeted Facebook intervention (3.7%) and the Smokefree website (1.7%) arms in the same study [5]. It is worth noting that study participants’ readiness to quit smoking at baseline spanned the full range of the Contemplation Ladder, from 0 to 10, suggesting that even sexual and gender minority young adults with very low motivation to quit may be willing to engage with, and may benefit from, an intervention that is designed for smokers at all levels of quit readiness. This was the case in the original Flexiquit trial, where a 29% quit rate was obtained even though 65% of the sample reported low readiness to quit at baseline [7].

In order to understand the impact of the EQQUAL program on ACT’s key theory-based change mechanisms, we included several measures of psychological flexibility, which generally showed modest theory-consistent improvements. The largest improvements were observed for the Values Questionnaire. Greater change on the Values Questionnaire may be due to the heavy emphasis on values in the first 2 sessions, with the treatment dosage of later acceptance-based content limited by less engagement with the latter sessions. Alternatively, it could be that the values-based action component of psychological flexibility precedes changes in other processes that take longer to develop in this new context of ACT for smokers across all stages of quit readiness, as has been observed in studies of ACT for other conditions [28].

This study had a number of limitations that should be considered when interpreting the results. Primary limitations stem from the small sample size and that causality cannot be determined from a single-arm pilot trial; therefore, conclusions about EQQUAL’s efficacy or impact on theory-based change mechanisms are tentative and require evaluation in a larger, controlled clinical trial. Due to the small sample size, representativeness and generalizability are limited. For example, although 25% of the sample identified as a racial or ethnic minority, not every race and ethnicity could be represented. Another limitation concerning the generalizability of findings is that the data were collected during the early period of the COVID-19 pandemic, which may have impacted participants’ perceptions of the EQQUAL program and their willingness and ability to quit smoking. Based on the COVID-19-related questions that we included in the baseline and follow-up surveys, there were mixed findings regarding the pandemic’s impact on smoking and motivation to reduce or quit, with some participants reporting little or no impact, some increasing their smoking, and some decreasing their smoking or experiencing increased motivation to reduce or quit smoking. Overall, the reported intensity of smoking at baseline (ie, averaging 7.6 cigarettes per day) is not atypical, as a majority of young adults smoke less than one-half pack (ie, less than 10 cigarettes) per day [29]. Another limitation is that it was not feasible in this pilot trial to conduct the testing that would allow us to biochemically verify smoking abstinence among participants who used other nicotine and tobacco products (eg, e-cigarettes). In future work, we plan to use a multimethod approach combining remote carbon monoxide monitoring with cotinine testing to address this limitation.

In spite of the study’s limitations, this work is significant and innovative in 5 key respects: (1) It focuses on a tobacco-related health disparities group that has been underserved in health-related research to date and in tobacco treatment research in particular. (2) It is the first evaluation of a self-guided digital cessation treatment for sexual and gender minority young adults, maximizing scalability and addressing sexual and gender minority youths’ desire for a program targeted to their unique needs and challenges. (3) It applies a novel treatment approach and advances the science of ACT for tobacco cessation by testing its effectiveness for tobacco users at all stages of readiness to quit rather than only among those who are ready to quit. (4) The self-guided, digital format makes EQQUAL readily accessible to sexual and gender minority young adults who lack health insurance and who have low engagement and quit rates with currently available public health interventions for cessation. (5) Using avatars and interactive games as engagement strategies is substantially different than existing treatments. These strengths warrant continued development and evaluation of the EQQUAL program in a randomized controlled pilot trial.

## Appendix

Multimedia Appendix 1 EQQUAL participant flow diagram.

## Abbreviations

ACT: acceptance and commitment therapy
COVID-19: coronavirus disease 2019
EQQUAL: Empowered, Queer, Quitting, and Living program
PPA: point prevalence abstinence
