# Supplementary material for: An Avatar-Led Digital Smoking Cessation Program for Sexual and Gender Minority Young Adults: Intervention Development and Results of a Single-Arm Pilot Trial
Source: JMIR Form Res. 2021 Jul 30;5(7):e30241. doi: 10.2196/30241 (PMC8367141; doi:10.2196/30241)
Supplement: Multimedia Appendix 1 [file formative_v5i7e30241_app1.docx]

Multimedia Appendix 1. EQQUAL Participant Flow Diagram

Ineligible: 43

Smokes <1 cigarette per day over the last 30 days: 8

Does not have the ability to stream video: 8

Not between 18 and 30 years of age: 7

Does not meet sexual or gender minority status: 6

Receives current treatment for smoking cessation: 6

Does not have use of text messaging: 6

Participated in other FH studies: 5

Does not have weekly internet access: 3

Payment form not OK: 3

Here for someone else: 2

In prison: 2

Not willing to complete surveys: 2

Does not reside in the US: 2

Other household member participates in this study: 1

Does not have access to weekly email: 1

Not comfortable reading in English: 1

Screened: 118

Consented: 26

Eligible:75

Did not confirm interest in the study via email or complete baseline survey and consent: 49

**Included in final endpoints**

Primary (utilization): 22 (n=18 with at least one log-in)

Primary (satisfaction): 15

Secondary (abstinence, missing=smoking): 22

Secondary (abstinence, complete case): 17 for self-report, 16 for biochemical confirmation

Secondary (quit readiness): 17

Secondary (psychological flexibility): 16

Secondary (perceptions of avatar): 15

**2-month Follow-up**

Lost to follow-up: 5

Withdrawn: 3 (suspected fraud)

Completed: 17

Sent cotinine test based on self-reported abstinence from all forms of nicotine: 6

Returned cotinine results: 6

Excluded due to suspected fraud: 1

Received access to the EQQUAL Program: 25

Enrolled: 25
